# Providing “a beam of light to see the gaps”: determinants of implementation of the Systems Analysis and Improvement Approach applied to the pediatric and adolescent HIV cascade in Kenya

**DOI:** 10.1186/s43058-022-00304-3

**Published:** 2022-07-16

**Authors:** Kristin Beima-Sofie, Anjuli D. Wagner, Caroline Soi, Wenjia Liu, Deanna Tollefson, Irene N. Njuguna, Emily Ogutu, Douglas Gaitho, Nancy Mburu, Geoffrey Oluoch, Peter Mwaura, Peter Cherutich, Laura Oyiengo, Grace C. John-Stewart, Ruth Nduati, Kenneth Sherr, Sarah Gimbel

**Affiliations:** 1 Department of Global Health, University of Washington, Seattle, USA; 2 Department of Child, Family & Population Health Nursing, University of Washington, Seattle, USA; 3 Research & Programs, Kenyatta National Hospital, Nairobi, Kenya; 4 Gangarosa Department of Environmental Health, Emory University, Atlanta, USA; 5 Network of AIDS Researchers in Eastern and Southern Africa, Nairobi, Kenya; 6 Ministry of Health, Nairobi, Kenya; 7 Department of Epidemiology, University of Washington, Seattle, USA; 8 Department of Pediatrics, School of Medicine, Seattle, USA; 9 Department of Medicine, University of Washington, Seattle, USA; 10 Department of Paediatrics and Child Health, University of Nairobi, Nairobi, Kenya; 11 Department of Industrial & Systems Engineering, University of Washington, Seattle, USA

**Keywords:** Consolidated framework for implementation research (CFIR), Implementation determinants, Pediatric, Adolescent, HIV, Health systems, Implementation science, Systems engineering, Cascade analysis, Flow mapping, Continuous quality improvement, Systems Analysis Improvement Approach (SAIA)

## Abstract

**Background:**

Children and adolescents living with HIV have poorer rates of HIV testing, treatment, and virologic suppression than adults. Strategies that use a systems approach to optimize these multiple, linked steps simultaneously are critical to close these gaps.

**Methods:**

The Systems Analysis and Improvement Approach (SAIA) was adapted and piloted for the pediatric and adolescent HIV care and treatment cascade (SAIA-PEDS) at 6 facilities in Kenya. SAIA-PEDS includes three tools: continuous quality improvement (CQI), flow mapping, and pediatric cascade analysis (PedCAT). A predominately qualitative evaluation utilizing focus group discussions (*N* = 6) and in-depth interviews (*N* = 19) was conducted with healthcare workers after implementation to identify determinants of implementation. Data collection and analysis were grounded in the Consolidated Framework for Implementation Research (CFIR).

**Results:**

Overall, the adapted SAIA-PEDS strategy was acceptable, and the three tools complemented one another and provided a relative advantage over existing processes. The flow mapping and CQI tools were compatible with existing workflows and resonated with team priorities and goals while providing a structure for group problem solving that transcended a single department’s focus. The PedCAT was overly complex, making it difficult to use. Leadership and hierarchy were complex determinants. All teams reported supportive leadership, with some describing in detail how their leadership was engaged and enthusiastic about the SAIA-PEDS process, by providing recognition, time, and resources. Hierarchy was similarly complex: in some facilities, leadership stifled rapid innovation by insisting on approving each change, while at other facilities, leadership had strong and supportive oversight of processes, checking on the progress frequently and empowering teams to test innovative ideas.

**Conclusion:**

CQI and flow mapping were core components of SAIA-PEDS, with high acceptability and consistent use, but the PedCAT was too complex. Leadership and hierarchy had a nuanced role in implementation. Future SAIA-PEDS testing should address PedCAT complexity and further explore the modifiability of leadership engagement to maximize implementation.

**Supplementary Information:**

The online version contains supplementary material available at 10.1186/s43058-022-00304-3.

Contributions to the literature

- Research is sparse about the determinants of implementation for multi-component, systems-focused implementation strategies.
- We identified quality improvement and flow mapping as core components of a multi-component, systems-focused implementation strategy and identified a component (the pediatric cascade analysis tool) that was too complex to be useful. We also identified mechanistic lessons about how these core components operated, utilizing a meta-theoretical framework, the Consolidated Framework for Implementation Research.
- We identified the nuanced and complex role of leadership engagement and hierarchy in implementing a systems-focused implementation strategy.
- These findings provide evidence of how complex strategies operate in low-resource settings and identify how contextual factors can influence implementation.

## Introduction

Children, adolescents, and youth living with HIV have poorer rates of HIV testing, treatment response, and virologic suppression than their adult counterparts. HIV treatment coverage for children and adolescents aged 0–15 was 53% vs 68% for adults globally in 2020 [1]. Additionally, each of these age groups has distinct HIV-related challenges at the individual and structural levels at each step of the HIV cascade [2–5]. The complexity of systems, changing guidelines, and heterogeneity between facilities serving these populations contribute to suboptimal adherence to guidelines for HIV testing and treatment. Historic and unequal relationships in data vacuuming and reporting up often leave front-line healthcare workers (HCWs) disconnected from the data they routinely collect [6].

Many health systems interventions and implementation strategies have addressed individual steps in the HIV cascade—HIV testing, antiretroviral therapy initiation, viral load monitoring, and suppression—but few attempted a systems approach to optimize the multiple, linked steps simultaneously. The Systems Analysis and Improvement Approach (SAIA) [7, 8] is a multi-component implementation strategy that combines three tools from systems engineering—cascade analysis [9], flow mapping, and continuous quality improvement. It was developed and tested in three African countries (Côte d’Ivoire, Kenya, and Mozambique) and was shown to reduce drop-off in the prevention of mother-to-child transmission of HIV (PMTCT) cascade [8]. SAIA has been adapted to diverse health settings and conditions, including family planning, cervical cancer screening, hypertension, mental health, malaria, opioid overdose reversal, and further scale-up within PMTCT [10–14].

A previous evaluation of the original SAIA approach applied to the PMTCT cascade was conducted to identify determinants—barriers and facilitators—of successful implementation. This evaluation revealed that a limited set of constructs from the Consolidated Framework for Implementation Research (CFIR) distinguished between high- and low-performing settings, including networks and communication, available resources, external change agents, executing, reflecting, and evaluating. The second series of constructs were weakly associated with high versus low performance (intervention source, relative advantage, complexity, tension for change, relative priority, goals, and feedback), mostly falling within the domains of inner setting, intervention characteristics, and process [15]. Identifying such determinants is useful in refining, adapting, and optimizing further expansion of implementation strategies, such as SAIA.

Our team adapted the original SAIA strategy to be specific to the pediatric, adolescent, and youth HIV care and treatment cascade; the adapted version (SAIA-PEDS) was piloted and demonstrated promising but heterogeneous impacts across the pediatric and adolescent HIV cascade in a quantitative evaluation [16]. In this qualitative evaluation, we assessed the implementation of SAIA-PEDS.

## Methods

### Study design and population

Between December 2018 and March 2019, we conducted a qualitative evaluation of the SAIA-PEDS implementation strategy, which had been piloted previously in 2018 at six facilities across four counties in Kenya (three were located in Nairobi County, one in Homa Bay County, one in Siaya County, and one in Kisumu County). We selected the facilities purposively to represent diversity in the level of services offered and size of the facility; there were two county hospitals, sub-county hospitals, and health centers [16]. The SAIA-PEDS multi-component implementation strategy aims to facilitate group problem solving with HCWs to test the changes at health facilities to reduce drop-off and prioritize steps for optimization in a particular cascade. The SAIA-PEDS strategy is an adapted version of the original SAIA strategy; detailed explanations of the various adaptations made to the strategy are described elsewhere [16]. Briefly, the largest adaptation was in the cascade analysis tool, which was adapted in partnership with stakeholders to be relevant to the pediatric and adolescent HIV cascade. In the pilot, we used a pre-post analysis of routine program data with 6 months pre- and 6 months post-implementation to assess the changes in HIV testing, linkage to care, antiretroviral treatment (ART), viral load (VL) testing, and viral load suppression for children and adolescents. In the quantitative evaluation, the SAIA-PEDS strategy was found to have a substantial and significant improvement on viral load monitoring and suppression, but no impact on HIV testing, linkage to care, or treatment initiation [16]. We used a purposive sampling strategy to recruit all HCWs at study facilities involved in the provision of pediatric or adolescent HIV services at the time of recruitment. All HCWs were approached in person. We aimed to have a higher cadre (facility matrons, in-charges) participants complete IDIs and lower cadre (nurses, counselors, peer counselors) participants complete FGDs to allow team members to speak freely without hierarchical pressure.

### Ethical approval

This study was reviewed and approved by the Kenyatta National Hospital/University of Nairobi Ethics and Research Committee and the University of Washington Institutional Review Board. All participants were ≥ 18 years of age and provided written informed consent for participation. County leadership was engaged during the implementation planning and permission-seeking process prior to study implementation and updated on study progress periodically.

### Theoretical framework

This evaluation was structured and analyzed using CFIR [17], a meta-theoretical model appropriate for assessing determinants of implementation. Qualitative question guides were developed by adapting the CFIR.org website questions across five domains: inner setting (e.g., compatibility, available resources), intervention characteristics (e.g., complexity, adaptability), process (e.g., planning, engaging, executing), outer setting (e.g., external policies and incentives), and characteristics of individuals (e.g., self-efficacy). Most questions focused on constructs within the inner setting and intervention characteristics domains, with fewer questions related to the other domains (Additional file 1: Appendix 1: Question guides with CFIR domains and constructs noted). The question guides were developed, refined, and piloted by the study team members (KBS, EA, ADW, SG, GO, NM, MM). One quantitative scale, the Shorter Adaptive Reserves Measure [SARM] (abbreviated 14-item scale from the Practice Adaptive Reserves scale [18]), was included as a quantitative survey that maps to the CFIR domain of inner setting, specifically to constructs related to team functioning and leadership engagement.

### Data collection

Following completion of written informed consent, HCWs completed the quantitative SARM survey and participated in either a single focus group discussion (FGD) or individual interview (IDI) at their facility. One FGD and 2–4 IDIs were conducted at each facility by a trained, experienced female Kenyan social scientist who was not involved in study implementation and had no prior relationship with study participants (EA). Only IDI and FGD participants were present during qualitative discussions with the interviewer, and participants were informed that information shared during discussions would be kept confidential from the SAIA-PEDS implementation study team. FGDs had between 5 and 10 participants and lasted an average of 97 min. IDIs lasted an average of 42 min. FGDs and IDIs were conducted in English, audio-recorded, and transcribed. To summarize the key concepts discussed, targeted debrief reports were written by the interviewer immediately following each FGD or IDI.

### Qualitative data analysis

Transcripts were analyzed using a directed content analysis approach [19]. The analysis team (KBS, CS, WL, ADW, SG) iteratively developed and refined a comprehensive codebook that was used to code all transcripts. Initial codebook development and refinement were done using debrief reports, followed by reviewing a limited set of transcripts representing each of the study facilities. Coding employed a primarily deductive approach, using CFIR construct codes from discussion guides, with construct definitions operationalized to study-specific design and context through transcript review. Codes represented CFIR constructs, were grouped into corresponding CFIR domains, and expanded to include detailed code definitions with inclusion and exclusion criteria and example excerpts. Each transcript was independently coded by one member of the analysis team and then reviewed by another member of the team to assess consistency with the initial code application and note discrepancies. All coding discrepancies were resolved through consensus meetings. Analysts also drafted and reviewed detailed memos for each transcript, noting key CFIR constructs that facilitated or hindered SAIA-PEDS implementation. Group discussions were used to identify common constructs influencing implementation at each facility and compare constructs and implementation experiences across facilities. Coding and data management were conducted in ATLAS.ti version 8 (Scientific Software Development GmbH), and queries and code co-occurrence tables were used to verify findings and identify quotes associated with specific CFIR constructs.

### Quantitative data analysis

DT analyzed the SARM quantitative items, conducting descriptive statistics of mean and standard deviation for the overall score and the individual score components both within and between facilities in R. ADW conducted Kruskal-Wallis rank tests for the overall score and individual score components using STATA version 14 (College Station, TX).

## Results

A total of 42 HCWs participated in 6 FGDs, and 19 HCWs participated in IDIs across six facilities. All HCWs had been involved in the SAIA-PEDS process. Interview participants primarily represented higher cadre HCWs (*n* = 13; 68%). Overall, HCWs reported a good understanding of the SAIA-PEDS tools, how they worked together and complemented one another, and how they improved the provision of pediatric and adolescent HIV services (Table 1).

I think each and every tool is important and it assists the other tools so that you work out your issues. I don’t think any is less important to use because if you use PedCAT it will help you have a good flow of the patients and at least see who is dropping where and where to bring up some change but again it even helps the CQI. They help each other so they have to work together. – Facility 2, FGD

**Table 1:** CFIR constructs, mechanism, use, and illustrative quotes for each SAIA-PEDS tool and the overall package

	**SAIA strategy overall**	**PedCAT**	**Flow mapping**	**Continuous Quality Improvement**
CFIR constructs	Relative advantage	Complexity	Compatibility	Networks and communication
Explained mechanism	Provided a structured approach to identify gaps in services	Not useful because not available in real time to inform decisions Too complex, difficult to use without additional training Obtaining data to populate the PedCAT was challenging	Illuminated specific gaps; intuitive Worked together with CQI	Provided targeted action plan, goal, objectives, and structure for their existing meetings Promoted a positive learning environment and safe space for identifying and discussing challenges and bringing forward mistakes Facilitated working together to solve problems as a group and ensure an overall better care environment
Relative use frequency		Rarely used	Used repeatedly in some clinics	Used repeatedly at most clinics
Illustrative quotes	“The three tools serve as [a] performance indicator on what we do, the whole work, the workload we have and the time frame, the planning of everything. If we inherit the three tools that means we improve our performance and the targets are met.” – Facility 6, FGD	“The PedCAT was a bit challenging and even up to now, I find it difficult to understand it well that I can use it to tell somebody else about SAIA. From the training, it should be [that] if I am faced with it, I can explain it to my neighbor, but I still find it a bit challenging on that.” – Facility 5, FGD	“The training helped us understand where our patients were getting lost before completing the process. It made us understand there were clients who were getting lost at the linkage point because of our flow map. So, it made us understand how to improve our flow map at the facility in a short time.” – Facility 3, FGD	“The meetings are very, very important, they have really helped us in improving our services. Honestly, we do learn a lot when we sit down as a group or a team, maybe there was a mistake somewhere, you admit it was a mistake but now, what is the way forward, what can you do so that this do not happen again, yes.” – Facility 2, Interview 3

### Relative advantage, implementation climate—tension for change, and implementation climate—relative priority constructs influenced overall perceptions about the SAIA—PEDS strategy

The majority of HCWs felt that the SAIA-PEDS strategy as a whole provided an advantage (intervention characteristics: relative advantage) when compared to existing processes because it provided a structured way to identify gaps in services provided and focused on a key population (children and adolescents) that was falling behind in reaching the “95-95-95” goals. SAIA-PEDS allowed HCWs to focus on the “right” challenges in the facilities and gave them the tools to identify specific solutions that would address those challenges, therefore providing structured direction and focus for improving the quality of services offered.

[I]t’s like flashing, giving a big beam of flashlight to see the gaps… they are basically being highlighted through this SAIA study and for me that one is something that went well. – Facility 6, Interview 1

Okay before the study, we were not able to know areas maybe where we have drop outs, and be able to rectify the areas, but after the study, now we have the tools and we are able to go through the tools and know the areas where we have drop outs and where maybe we can use the flow maps to get the areas where we can book clients who are missing out and be able to rectify the areas. – Facility 2, FGD

Prioritization influenced the implementation of SAIA-PEDS tools by the HCWs. Initially, some facilities lacked motivation for initiating changes (inner setting: implementation climate—tension for change), describing limited desire or pressure to make changes to existing processes. SAIA-PEDS was not seen as a priority by HCWs from these facilities when compared to existing work processes (inner setting: implementation climate—relative priority). However, continued implementation built evidence for why the strategy tools were important, improved confidence in HCW’s abilities to use tools, and eventually shifted HCW beliefs about the level of effort needed to routinely use SAIA-PEDS tools, therefore shifting prioritization and motivating implementation.

I came to realize those approaches didn’t need technical things and a lot of say, training or CMEs [continuing medication education sessions], these were just the same, same routine things we do, you know, and at first there was a lot of resistance. But later on people went on and they realized these were just simple things that we could do. – Facility 1, Interview 3

SAIA was a new thing, to begin with, of course we were wondering why are they pushing us… But now with its support, I would say SAIA, they gave us that support also, so with their support, then helping us to understand the importance like if these people are diagnosed early then you will not even need to keep them in your wards. – Facility 3, Interview 1

### Networks and communications and implementation climate—compatibility constructs were associated with the value placed on continuous quality improvement processes

HCWs believed that continuous quality improvement approaches supported improved communication between HCW teams (inner setting: networks and communications). The SAIA-PEDS strategy relied heavily on group problem solving. HCWs described how challenges to providing optimized pediatric HIV care and their identified solutions often transcended beyond a single department and required a strong network of support and good communication between departments to be successful. One team specifically noted that CQI meetings helped facilitate systems thinking by promoting testing changes across different departments.

[T]hat assignment cannot be done by one person, coz it’s inter-linking like all the departments… So, we have to sit down and agree. Like that one time we wanted modification of the flow of the patients, I couldn’t just do it on my own because I will interrupt with the services on the other side, so we had to sit down as a team and agree, will this work out, so we give it a try, if it goes well, we adopt it. – Facility 1, Interview 1

HCWs believed that CQI meetings and flow mapping processes were especially helpful in supporting team communication, improving service provision, and facilitating facility goal achievement. By promoting a positive learning environment and providing a safe space for identifying and discussing challenges and bringing forward mistakes, teams noted how CQI meetings were able to facilitate working together as a group to solve problems and ensure improved overall care.

We were able to bring people together; do you know there were even some departments where people never used to talk to each other? They were always busy you know, especially the lab. The lab people would always tell the CCC [HIV care clinic] people you are sending us too many people for viral load and what. But then this meeting brought people together in such a way that there was now cohesive… people came together as a team and they would talk and laugh. – Facility 1, Interview 3

The meetings, they are very important, they have really helped us in improving our services. Honestly, we do learn a lot when we sit down as a group or a team, maybe there was a mistake somewhere, you admit it was a mistake but now, what is the way forward, what can you do so that this [does] not happen again. – Facility 2, Interview 2

SAIA-PEDS was compatible with existing workflows and supported group ownership of ideas and solutions (inner setting: implementation climate—compatibility). CQI and flow mapping resonated with teams at all facilities and were compatible with the current workflows and values of the facility, aligning with the facility’s overarching goal of providing quality care and systems already in place to achieve this goal. HCWs noted compatibility between their non-SAIA-PEDS jobs and their SAIA-PEDS team roles. One facility had a strong SAIA-PEDS team identity, making a WhatsApp group specifically for their coordination efforts.

You know, the good thing is this is something that blended already in what they were doing so it did not require any more resources, anything different that they would be able to require, just the knowledge they had and the implementation process, so they were doing their ordinary job as they implement SAIA. So, it was one stop shop. – Facility 2, Interview 3

Many teams believed that CQI helped amplify the impact of their existing meeting structure by helping to prioritize problems and give structure to iteratively trying new ideas, specifically focusing on adolescents and children, providing an “opportunity to broaden participation in problem solving.”

[T]he importance of this meeting is that you will not be sitting down making recommendations and come back again to look at those recommendations and talk about them again and again, when you meet this time you have resolutions, you make recommendations and then next time you sit you will be able to review and see we recommended this in the other meeting, what have we been able to achieve, what haven’t we been able to achieve, what made us not achieve this percentage that we did not, how do we re-strategize to be able to achieve it this time round. – Facility 2, Interview 3

The data collected during SAIA-PEDS also matched the data on which HCWs were evaluated for performance and was used to help encourage HCWs to adopt new procedures and practices to improve care.

I used this method of using data, so when you use data, and show this is where we were and this is now where we are, then that particular worker will see that really they are not doing well in terms of work and of course the government wants them also to sign a performance contract, so of course it weighs down on the performance contract of that particular staff. So, I felt this was a good approach to use. – Facility 1, Interview 3

Many facilities reported that aligning CQI with the UNAIDS 95-95-95 goals made the goals seem more achievable. Among the one team with a strong culture of quality improvement pre-intervention, CQI and flow mapping supported the team to focus on later steps of the cascade (e.g., viral load monitoring), because they had already worked to optimize earlier steps.

HCWs felt that flow mapping and CQI meetings were also compatible with their goal to meet patient needs by improving service delivery. HCWs described implementing changes that helped them improve linkage to care by physically walking patients to services, identified bottlenecks to rapid return of viral load results by implementing detailed tracking systems, and providing data-informed adherence counseling for children and adolescents with high viral loads based on data being available.

### Complexity, readiness for implementation—access to knowledge and information, and engaging—external change agents constructs provided rationale supporting underlying limited use of the PedCAT

Universally, HCWs found the PedCAT overly complex (intervention characteristics: complexity), difficult to use, and not as useful as the other SAIA-PEDS tools. With the exception of one facility, HCWs felt that obtaining data to populate the PedCAT was challenging, particularly in facilities with poor communication between team members and exceptionally high patient volumes.

[T]he outpatient department, it’s busy all the time, so for you to get hold of that under five register is not easy… So, they had a challenge of getting those registers because OPD [outpatient department] is busy full time not unless they come at night, but at night people need to go to their houses. – Facility 2, Interview 1

One facility described how the data sources used to fill the PedCAT were not well understood, generating confusion around how to derive the numbers and a lack of confidence in the tool. At facilities where data access was less of a barrier, HCWs reported that collating data for the PedCAT improved overall data quality and led to improved service delivery.

HCWs suggested that more training on the use of the PedCAT (inner setting: readiness for implementation—access to knowledge and information) could improve uptake. Specifically, HCWs noted that training to understand formulas embedded within the PedCAT, more time to accrue indicators (particularly viral load data), and access to an electronic version of the tool would have made the data collection process less complex/confusing.

[B]earing in mind that it was being printed only, so, you see, there are some figures you input there to calculate and give the percentage. So, we could not really understand, ‘where is this coming from, where is this coming from?’... because it is already filled and it’s printed. So we need more of a visualized PedCAT, probably we could interpret it well than get it automated. …So, I think that was the most challenging thing. – Facility 6, FGD

HCWs at all facilities noted that a 1-day initial training was too short, and many teams wanted a longer period of mentorship from the SAIA-PEDS team to learn and get things up and running.

What we can say is that the training one, was too short because we were given only one day of training but looking overall on the requirements, the training that ought to have taken a longer time maybe even a week so that we could run all over the program, but it was condensed within one day. That was not sufficient, right. – Facility 3, FGD

In facilities with challenges in launching SAIA-PEDS, having only some team members trained was interpreted as exclusionary and limited enthusiasm for participating in SAIA-PEDS.

I think not everybody was being involved so some people might have felt that they are left out, that this is a project of some few individuals whereby if you go towards or to rectify the gaps, they just think it is your project, yeah. – Facility 6, FGD

Facilities that noted having challenges engaging with the SAIA-PEDS team continued to view the strategy as something external (process: engaging—external change agents) and were less likely to describe continued use after the trial ended. In contrast, HCWs at facilities that felt engaged with the SAIA-PEDS team more often noted compatibility between the goals of the strategy and those already existing at the facility, described feeling ownership of the strategy, and noted an easy integration of SAIA-PEDS into routine practice at the facility.

… what we believe in is that once we have a project in the hospital, we treat it as ours, so that we don’t have to say it is their project. So, we supervise them as our project so that you try to own it and after exit you can take [it] up. So, we try to institutionalize the programs that are being done in the hospital so it is always part of our duty… – Facility 5, Interview 1

### Readiness for implementation—available resources, engaging-formally appointed internal implementation leaders, and engaging-champions constructs positively affected overall implementation

Although facility leadership was universally reported to be strong, there was variability in specific examples of how that leadership manifested. HCWs from facilities that described more challenges with implementation reported that leadership was supportive of SAIA-PEDS (process: engaging—formally appointed internal implementation leaders) but had few specific examples of how leadership engaged, encouraged, and supported implementation. In contrast, HCWs from facilities with fewer challenges with implementation provided many specific ways in which leadership brought enthusiasm, dedicated physical space and time, and arranged backup leadership coverage. HCWs from these facilities described how leadership integrated incentives into supportive management structures that encouraged innovation and recognized appreciation for individual work in SAIA-PEDS by coworkers.

I felt appreciated how, one, even just being given that opportunity to come and give my opinion or sit in the meetings time and again, I feel that was a good form of appreciation and also the fact that they were also willing to listen to our advice or to our input, yeah, that is also ... a way of appreciation. – Facility 4, Interview 4

One facility noted how changes in leadership’s priorities hindered implementation of SAIA-PEDS when other programs were deemed more urgent and thus prioritized over SAIA-PEDS, given the limited resources and time.

There was competing interest we realized… initially we had a plan in line with what SAIA PEDS were interested in majorly improving HIV services of peds and adolescents but again when we presented that later it was changed by the leadership of the hospital to something else which was not really much in what SAIA PEDS support so there was competing interest between the SAIA team [in the facility] and the hospital leadership that made [it] not succeed so much. – Facility 3, FGD

While hierarchy and set leadership structure acted as a barrier to implementation in some settings, it facilitated implementation in others. In settings where hierarchy was a barrier, slow movement in approval from in-charges and diffuse responsibility across team members seemed more common, with blame put on individuals for failing to complete certain responsibilities. Lower cadre HCWs from one facility reported feeling disempowered and told it was “not their place” to introduce ideas during CQI meetings. In settings where hierarchy and set leadership structure acted as a facilitator, in-charges asked for updates frequently and used those updates to act as change agents to reinforce and keep the momentum for change, rather than slow processes down.

I think he appreciates [my work], because always, he would ask all the time, ‘what do you need, how can I help you?’ You see, that one motivates you, that he is part of the quality issues. – Facility 5, Interview 2

Internally, within clinic teams, champions helped facilitate fewer implementation challenges (process: engaging—champions). Facilities with fewer implementation challenges saw those who were trained by the SAIA-PEDS team as champions, responsible for training others in their facility. Champions were represented by different individuals and took on different roles. One facility with few implementation challenges created a specific SAIA-PEDS team lead and centralized team for moving the SAIA-PEDS process forward. Overall, champions were recognized as those who were motivated, had passion, and ensured implementation activities were accomplished.

The nurse manager was always there for the meeting, because when somebody is there for the meeting, it means he is ready and having a passion to do that. They were always committed. When we tell them there is a meeting, they are there. Whenever there were assignments, they were there….So, they were committed. – Facility 1, Interview 2

Time availability and workload of HCWs (inner setting: readiness for implementation—available resources) created feasibility challenges in implementing SAIA-PEDS. In facilities describing challenges with feasibility, teams reported incompatibility between existing workload and needs of the strategy, specifically the frequency and duration of the CQI meetings. One of the biggest challenges HCWs identified with SAIA-PEDS was the time commitment required to attend the meetings or to implement the procedures identified during CQI meetings. HCWs described an internal struggle between wanting to improve processes and also recognizing that meetings and activities increased their workload. HCWs felt many competing interests for their time, especially related to providing clinical care. Staff shortages, and burnout of current staff, prevented some gaps or solution plans from being addressed.

Time is what I am trying to tell you is a challenge. But you know, they say create time. It takes a lot of sacrifice. For me to attend that meeting, it means I have left my docket, so I either have to come in and work so much to catch up for the day or set aside some work so that I set some little time. – Facility 6, Interview 1

… at times this place had so many meetings, the SAIA meeting is coming, Wednesday is usually the day, we see our pediatrics on Wednesday so we book them on Wednesday and because most of the time it is the day we put our CMEs [continuing medical education sessions], it is the day we have any other meetings so at the end of the day we feel burdened that meetings are too many … So, sometimes we were like feeling a bit harassed. – Facility 4, Interview 1

Universally, teams wanted additional or different compensation for engaging in the SAIA-PEDS process to acknowledge the extra efforts that they took in implementing additional work activities. One team reported they liked that SAIA-PEDS did not have additional costs, because if it required additional resources other than time, that would have been a non-starter. Another facility commented that training additional HCWs could have reduced the demand for individual’s time, by spreading out implementation duties among more people.

Despite the increased workload, SAIA-PEDS processes were valued because they allowed the facilities to address challenges that interfered with providing the highest quality care for patients. SAIA-PEDS was adopted as part of clinic culture going forward at a few facilities. SAIA-PEDS helped shift perspectives about the value added by continued review and adaptation of facility processes. HCWs who participated realized that they do not need to make just one change and be done but can instead repeat this process continually, facilitating longer term adoption. In addition, HCWs recognized the value that SAIA-PEDS tools can bring to other health issues outside of pediatric and adolescent HIV, noting the transportability of these tools.

### Shorter adaptive reserve measure (SARM)

**Fig. 1:**
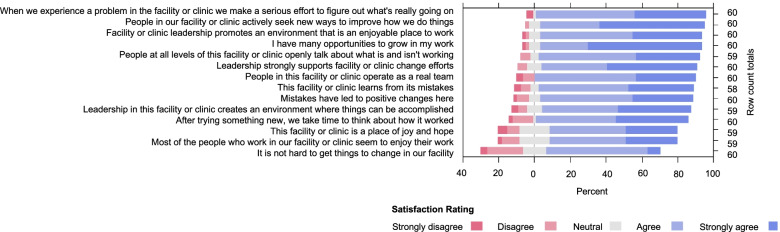
Distribution of responses to the shorter adaptive reserve measure

In addition to the qualitative assessments of determinants, we additionally assessed the inner setting using the SARM; 60/61 participants completed this quantitative survey. Among the 14 items in this tool, all items had a majority of the 60 respondents selecting either “strongly agree” or “agree,” with mean Likert scores for the items ranging from 3.4 to 4.5 (Fig. 1). The items with the highest mean scores were “people in our facility/clinic actively seek new ways to improve how we do things” and “I have many opportunities to grow in my work” (both mean 4.5). The item with the lowest mean score was “It is not hard to get things to change in our facility” (mean 3.4). The overall scores between the 6 facilities differed significantly, ranging from 3.8 to 4.5 (*p* = 0.006 by Kruskal-Wallis; Table 2). Among the 14 items, 5 were significantly different between facilities (*p* < 0.05 by Kruskal-Wallis; Table 2).

**Table 2:** Individual item scores for each facility

Question	All facilities (***N*** = 60)		Facility 1 (***n*** = 8)		Facility 2 (***n*** = 9)		Facility 3 (***n*** = 9)		Facility 4 (***n*** = 12)		Facility 5 (***n*** = 9)		Facility 6 (***n*** = 13)		***p***-value*
	Mean	(SD)	Mean	(SD)	Mean	(SD)	Mean	(SD)	Mean	(SD)	Mean	(SD)	Mean	(SD)	
**1. Mistakes have led to positive changes here**	4.1	(0.9)	3.1	(1.2)	4.2	(0.7)	3.9	(1.3)	4.3	(0.6)	4.3	(0.7)	4.4	(0.5)	0.208
2. I have many opportunities to grow in my work.	4.5	(0.8)	4.5	(0.8)	4.2	(1.1)	4.2	(1.3)	4.8	(0.4)	4.4	(0.7)	4.5	(0.7)	0.665
3. People in our facility or clinic actively seek new ways to improve how we do things.	4.5	(0.7)	4.3	(0.7)	4.4	(0.7)	4.4	(0.7)	4.3	(1.0)	4.6	(0.5)	4.8	(0.4)	0.323
4. People at all levels of this facility or clinic openly talk about what is and is not working.	4.2	(0.8)	4.0	(0.5)	3.7	(1.0)	4.1	(0.9)	4.5	(0.7)	4.2	(0.4)	4.5	(0.7)	0.103
**5. Leadership strongly supports facility or clinic change efforts.**	4.3	(0.8)	4.4	(0.7)	3.8	(1.1)	4.1	(0.8)	4.2	(1.0)	4.3	(0.5)	4.9	(0.3)	**0.023**
6. After trying something new, we take time to think about how it worked.	4.1	(1.0)	3.6	(1.2)	4.3	(0.5)	4.1	(0.9)	3.9	(1.2)	4.6	(0.5)	4.1	(1.3)	0.501
7. Most of the people who work in our facility or clinic seem to enjoy their work.	3.9	(1.0)	3.3	(1.0)	3.4	(0.7)	3.7	(1.2)	3.8	(1.3)	4.3	(0.5)	4.4	(0.7)	**0.048**
**8. This facility or clinic is a place of joy and hope.**	3.8	(1.1)	3.0	(1.2)	3.2	(1.1)	3.9	(0.9)	4.0	(1.4)	4.3	(0.7)	4.2	(0.6)	**0.031**
9. This facility or clinic learns from its mistakes.	4.1	(1.0)	3.3	(1.4)	4.1	(0.6)	4.1	(0.8)	4.1	(1.4)	4.6	(0.5)	4.3	(0.5)	0.208
**10. Facility or clinic leadership promotes an environment that is an enjoyable place to work.**	4.2	(0.8)	3.9	(0.6)	3.9	(0.8)	4.1	(0.8)	4.0	(1.0)	4.6	(0.5)	4.8	(0.4)	**0.008**
11. People in this facility or clinic operate as a real team.	4.1	(1.0)	3.8	(1.5)	3.8	(0.7)	3.7	(1.0)	4.0	(1.0)	4.7	(0.5)	4.5	(0.5)	**0.018**
12. When we experience a problem in the facility or clinic, we make a serious effort to figure out what is really going on.	4.3	(0.8)	4.4	(0.5)	3.8	(1.2)	4.3	(0.5)	4.3	(1.1)	4.3	(0.5)	4.5	(0.5)	0.528
13. Leadership in this facility or clinic creates an environment where things can be accomplished.	4.1	(1.0)	4.1	(0.6)	3.9	(0.8)	3.8	(1.4)	3.8	(1.4)	4.3	(0.5)	4.7	(0.6)	0.125
14. It is not hard to get things to change in our facility	3.4	(1.0)	3.5	(0.9)	3.2	(1.0)	3.3	(1.2)	3.3	(1.1)	3.6	(0.9)	3.6	(1.0)	0.915
**Summary**	**4.1**	**(0.3)**	**3.8**	**(0.5)**	**3.9**	**(0.4)**	**4.0**	**(0.3)**	**4.1**	**(0.4)**	**4.4**	**(0.3)**	**4.5**	**(0.3)**	**0.006**

*Assessed using the Kruskal-Wallis *H* test, a rank-based, non-parametric test

The qualitative and quantitative results revealed heterogeneity between facilities in the strength of leadership engagement. Two of the 3 SARM items that addressed leadership engagement (“facility or clinic leadership promotes an environment that is an enjoyable place to work,” “leadership strongly supports this facility or clinic change efforts”) were significantly different in the facility-specific scores (*p* = 0.008 and 0.023, respectively). The three facilities with the highest scores on these two quantitative items also had the most detailed qualitative descriptions of the specific ways in which leadership supported the implementation of SAIA-PEDS, indicating concordance between the qualitative and quantitative data.

## Discussion

Within this predominately qualitative implementation study grounded in CFIR, we evaluated the core determinants of implementing the SAIA-PEDS strategy at 6 facilities across Kenya. Overall, HCWs perceived the SAIA-PEDS strategy as acceptable, and felt that the three tools complemented each other well and provided a relative advantage over existing practice processes. They believed CQI and flow mapping tools were compatible with existing workflows, resonated with team priorities and goals, improved HCW communication, and provided a safe environment to engage in group problem solving. In contrast, the PedCAT was found to be overly complex, with too little training given to understand the inner workings required for interpretation, limiting utility for HCW teams and impact across facilities. Aside from the tools of the strategy, teams found that facility leadership was a major determinant of implementation success. Whereas some facilities reported generally supportive leadership with few specifics, others described in detail how their leadership was engaged and enthusiastic, dedicated time and space, and created recognition systems for HCWs involved in SAIA-PEDS implementation; these messages were echoed in the quantitative surveys that assessed leadership and facility inner setting function.

The CQI and flow mapping elements of the SAIA-PEDS strategy fit well into the existing plans, values, and goals of the facilities. Facilities aimed to achieve the UNAIDS 95-95-95 goals for HIV testing, treatment, and virologic suppression, and HCWs working within the facilities aimed to deliver quality services to their clients. Both aligned well with the goals of the CQI activities. While many facilities had already existing meetings for review and improvement, the SAIA-PEDS CQI meetings gave HCW teams a structured process for addressing existing goals through group decision-making. These findings align with previous studies noting the impact of CQI as a tool for directing locally driven improvement in resource-limited settings across the HIV testing, treatment, and suppression cascade [20–22], as well as the theoretical models informing the SAIA-PEDS strategy (Donabedian’s model of quality in healthcare [23] and Deming’s theory of profound knowledge [24]). These flexible tools are useful to teams with diverse cadres, foci, and settings. In the CFIR-based evaluation of the original SAIA-PEDS strategy, both flow mapping and CQI were identified as core components of the SAIA-PEDS strategy [15].

In contrast to the CQI and flow mapping elements of the SAIA-PEDS strategy, the PedCAT was found to be highly complex, which resulted in limited utility. HCWs reported that data were unavailable in real time for decision-making due to the complexity and absence of data sources, making it difficult to collate PedCAT inputs and interpret them. Compared to the CAT for other SAIA adaptations, such as family planning or cervical cancer screening, PedCAT was more complex due to the many sequential steps involving numerous data sources and formats for the pediatric HIV care cascade. Multiple entry points, heterogeneous age cutoffs between departments, separate physical buildings, and multiple data formats (paper registers, paper files, and electronic files) limit the ability to use pre-aggregated data to easily populate the tool [9]. In the original SAIA strategy applied to PMTCT systems, the CAT was found to be overly complex in one setting with low volume, but useful in other settings [15], noting the tension between complexity and utility for this specific tool. In other SAIA adaptations, simplification, automation using a newly created electronic system, and mobile operation of the CAT have increased acceptability, perceived utility, and utilization of this tool [25]. Ultimately, refinement in the way that routine data are aggregated and reported across the pediatric and adolescent HIV cascade would likely impact the usefulness of the PedCAT.

Beyond the elements of the SAIA-PEDS strategy, the role of supportive leadership and hierarchy were complex determinants of implementation in our study, as well as the original SAIA evaluation [15]. In the original SAIA evaluation, leadership was described by multiple participants as crucial but did not emerge as a distinguishing factor between high and low performing facilities [15]. While all facilities in our study described leadership support for SAIA-PEDS, nuances in language between different teams highlighted how specific roles and actions may be more likely to promote the implementation of new strategies. Less successful teams described leadership support vaguely, while more successful teams gave concrete examples of the ways in which their leaders supported the SAIA-PEDS process. The quantitative scores on the SARM, intending to quantitatively measure leadership engagement and facility function, aligned with the qualitative findings between facilities; this suggests that while constructs related to leadership engagement may be challenging to measure, this quantitative tool did have discriminatory power in this setting. In a review and psychometric evaluation of quantitative measures that address inner setting CFIR constructs, the leadership engagement items (reflected in SARM) were found to have good structural validity, reliability, and discriminant validity [26].

In our evaluation, the CFIR constructs of networks and communication, available resources, relative advantage, complexity, tension for change, relative priority, and goals and feedback were identified as common determinants. These aligned mostly with those identified in the original SAIA evaluation, in which 5 constructs were strongly associated with high versus low performance (networks and communication, available resources, external change agents, executing, and reflecting and evaluating), and 6 were weakly associated with high versus low performance (intervention source, relative advantage, complexity, tension for change, relative priority, and goals and feedback), mostly falling within the domains of inner setting, intervention characteristics, and process [15]. We also identified compatibility and readiness for implementation as crucial determinants, both falling within the inner setting domain.

Our study was limited in that we were not able to distinguish between high- and low-performing facilities in our sample of pilot facilities, limiting our ability to identify distinguishing determinants, as was done in the original SAIA evaluation. It would have been more meaningful to identify determinants of specific implementation outcomes—such as acceptability, adoption, or fidelity—however, when we designed the evaluation of this pilot, we were unable to incorporate measurement of these outcomes and therefore identify specific determinants. Future trials of the SAIA PEDS multi-component implementation strategy should include well-designed and robust evaluations that assess the determinants of specific implementation outcomes. However, our study benefited from utilizing a strong meta-theoretical framework, the CFIR [17], which has been used broadly in implementation science and increasingly in low- and middle-income countries [27], allowing comparison with other studies. Finally, we used a robust coding and analytic approach with multiple independent analysts and were able to compare our study with the original SAIA strategy in a different context.

## Conclusion

In this evaluation of SAIA-PEDS, we identified CQI and flow mapping as core components with high acceptability and consistent use. The PedCAT was perceived as too complex for regular use, reflecting the complexity of the pediatric and adolescent HIV care cascade and related data systems. Critical determinants were similar in this adapted SAIA-PEDS to the original SAIA strategy, with additional emphasis on the nuanced role of leadership and hierarchy. If more broadly implemented, the SAIA-PEDS strategy should address PedCAT complexity and further explore the modifiability of leadership engagement to maximize implementation.

## Supplementary Information

**Additional file 1.** SAIA-PEDS Focus Group Discussion Question Guide.

**Additional file 2.** SAIA-PEDS In-Depth Interview Question Guide.

## Data Availability

The datasets used during the current study are available from the corresponding author on reasonable request.
