# Supplementary material for: Providing “a beam of light to see the gaps”: determinants of implementation of the Systems Analysis and Improvement Approach applied to the pediatric and adolescent HIV cascade in Kenya
Source: Implement Sci Commun. 2022 Jul 16;3:73. doi: 10.1186/s43058-022-00304-3 (PMC9287987; doi:10.1186/s43058-022-00304-3)
Supplement: Supplementary file 1 — Additional file 1. SAIA-PEDS Focus Group Discussion Question Guide. [file 43058_2022_304_MOESM1_ESM.docx]

**SAIA-PEDS Focus Group Discussion Question Guide**

**Instructions:**

The following is a guide. Try to ask all the questions below in the order given, but it is more important to maintain the flow of discussion. Suggested probes have been included. Start with the following introductory script:

***************************************************************************************************************************************

*Hi, my name is ____________. Thank you for agreeing to participate in the focus group discussion today. I am interested in understanding your thoughts, experiences and opinions about doing the SAIA intervention in your facility. I will ask you questions that you are free to answer in any way you wish. Feel free to elaborate on any of your points. If a question is unclear to you, please feel free to ask me to explain it.*

*It is important that we respect everyone in the focus group. Everyone is free to express his/her opinion without judgment from anyone else. It is important that the topics we discuss and the things people share in the focus group remain confidential. We should all respect each other’s privacy and feel free to discuss anything in the group.*

*I would like to record the discussion so I don’t miss anything that you say. I will not include any of your names on any documents or in the recording. Your answers will be kept confidential, which means we will keep what you say private from others. Is it okay if I record our discussion?* [Wait for the participants to give verbal consent to recording]

*Before we start I would like to remind you that there are no wrong answers in this discussion. We are interested in knowing what you think, so please feel free to be open and share your point of view. We hope you can help us understand what did and did not work in the SAIA PEDS intervention so that we can make changes in the future. Your comments about what did not work are just as helpful as your comments about what did work. It is very important that we hear your opinion. You do not have to answer all the questions. If you want to stop the discussion or leave at any time, just let me know.*

*As we get started, I want to remind us of the names of some tools so that we are all together in understanding. “****SAIA****” is an intervention that your facility participated in to improve HIV testing, treatment, and virologic suppression for children and adolescents. SAIA had 3 tools that you used. The first tool was the “****PedCAT”*** *[show picture of PedCAT], which showed your facility’s monthly data for HIV testing, linkage to care, ART initiation, viral load testing, and viral load suppression. The second tool was making “****flow maps”*** *[show picture of a flow map] of how patients move through your facility for services. The last tool was the process of testing small changes in clinic operations, sometimes called workflow modifications; we called this continuous quality improvement, or “****CQI****”. Together, SAIA used the* ***PedCAT****,* ***flow mapping****, and* ***CQI****.*

*Today, I will ask your opinions and thoughts about the SAIA training, using the tools, and changes in your facility.*

***************************************************************************************************************************************

First, let’s discuss the **training** that you attended where you learned how to implement the SAIA tools. As a reminder, during this training, you drew flow maps, practiced interpreting data from the PedCAT about the number of people who complete each step in HIV testing and treatment, and practiced testing small changes with paper airplanes using CQI.

1. Thinking about this **training**, how do you feel that the training prepared you and your colleagues to do the SAIA process and improve care for children and adolescents in your facility? (CFIR domain and construct: Inner Setting: Readiness for Implementation: Access to Knowledge & Information) *(~10 minutes)*
   - What were the positive aspects of training? Negative?
   - Which of the tools did you feel most comfortable using after the training? Least comfortable?
   - What could we have done better?

Next, let’s discuss the **meetings** that you attended at your facility with a SAIA team member where you brainstormed, planned, and tested small changes. As a reminder, during these meetings, either [insert name of Nancy, Maina, Geoffrey, or Dr. Gaitho] visited your facility, you reviewed your flow maps of how patients move through steps in your facility, you interpreted your PedCAT, and you planned testing small changes. These meetings started out once a week and then reduced to once every month or two.

1. Thinking about these **meetings**, how do you feel that the meetings enabled you and your colleagues to use the SAIA tools and improve care for children and adolescents in your facility? (CFIR domain and construct: Inner Setting: Readiness for Implementation: Access to Knowledge & Information) *(~10 minutes)*
   - What were the positive aspects of the meetings? Negative?
   - What would you say about the frequency of these meetings? Why?
   - Your team had visits for a total of 6 months. Should this period have been longer or shorter? Why?
   - Which tools do you remember using the most? The least?
   - What could we have done better?
2. Next, let’s discuss the SAIA **tools**, PedCAT, flow mapping, and CQI and what made them easy and hard to use *(probe about one tool fully before proceeding to the next tool) (~25 minutes)*
   - In your own words, what was the purpose of [insert tool name]?
   - When did you actually use [insert tool name]? Did this change over time? (CFIR domain and construct: Innovation characteristics: adaptability)
   - What were the challenges of using [insert tool name]? (CFIR domain and construct: Innovation characteristics: complexity)
   - [*only for CQI*] How did you find tracking or counting data or information on this tool? (CFIR domain and construct: Innovation characteristics: complexity)
   - How did you find using [insert tool name]? Was it complicated? (CFIR domain and construct: Innovation characteristics: complexity)
   - What changes do you think should be made to [insert tool name] so that it can work effectively in your facility? What are the parts of [insert tool name] that should not be changed? (CFIR domain and construct: Innovation characteristics: adaptability)
   - Have you continued to use [insert tool name] at your clinic in any way since the study has ended?
3. In your own words, how were the SAIA tools related to one another? How do they help, or not help, link different services in your facility to one another? How is this similar or different from the way you have made changes in your facility in the past? (CFIR domain and construct: Innovation characteristics: complexity) *(~5 minutes)*
4. Think about one **specific small change** (workflow modification, micro-intervention) that you tested during SAIA, either successful or unsuccessful. Can you describe what was tested and why your team chose to test this? (CFIR domain and construct: Inner setting: Implementation climate: learning climate) *(~10 minutes)*
   - What plan did you make? How did you make this plan to test this specific change? (CFIR domain and construct: Process: planning)
   - What helped make it successful/fail? (CFIR domain and construct: Inner setting: Implementation climate: learning climate)
   - Were people happy with the outcome? (CFIR domain and construct: Inner setting: Implementation climate: learning climate)
   - Who did your team feel the need to consult or get approval from before you could test this specific change? Why did they need to give permission before this change was tested? (CFIR domain and construct: Inner setting: Implementation climate: learning climate & leadership engagement)
   - Were there any changes that you thought might be good to test, but for which you did not get the opportunity to test? What happened in this case? (CFIR domain and construct: Inner setting: Implementation climate: learning climate & leadership engagement)
   - What was the role of the SAIA study team member (Nancy, Geoffrey, Maina, or Dr. Gaitho) in making these plans and testing small changes?

Next, let’s discuss the whole **process** of SAIA, that means using all of the tools together to make changes in HIV testing and treatment for children and adolescents

1. What kinds of things made it easy or difficult to implement this intervention in your facility? *(~30 minutes)*
   - How well did SAIA fit with your existing work processes and practices in your facility? (CFIR domain and construct: Inner setting: Implementation Climate: compatibility)
   - How did the location of service delivery points make it difficult or easy to use the SAIA tools? (CFIR domain and construct: Inner setting: structural characteristics)
   - How did the way duties and responsibilities are divided between different people make it difficult or easy? (CFIR domain and construct: Inner setting: structural characteristics)
   - At your facility, are you trying to reach 90-90-90 HIV care goals? Are you trying to reach any other large, national goals for children and adolescents? Which ones? How do you think SAIA related to these goals, either helping you reach them or preventing you from reaching them? (CFIR domain and construct: outer setting: external policies & incentives)
   - Think about other important activities or programs at your facility for supporting children and adolescents living with HIV. How important is SAIA compared to those other programs? (CFIR domain and construct: inner setting: implementation climate: relative priority)
   - Do you think there is a strong need for the SAIA intervention at your facility? Why or why not? Do you think your colleagues and superiors feel that there is a strong need for the SAIA intervention? Why or why not? How did you get to know? (CFIR domain and construct: inner setting: implementation climate: tension for change)
   - What kinds of resources did you need to do the SAIA intervention? Did you have all of the resources that you needed? Why or why not? (CFIR domain and construct: inner setting: readiness for implementation: available resources)
     1. Human resources, staffing, finances, space, free time or released time

Now let’s talk about **leaders and champions** at your facility. We are interested in all kinds of people who have influence, the formally appointed leaders of the facility, the informal leaders in your facility, champions for specific patient populations or activities, leaders in your local area that are not part of your facility but have influence here, or even just the informal leaders that someone turns to at your facility when they have an important decision to make. (CFIR domain and construct: process: engaging opinion leaders, formally appointed implementation leaders, champions, external change agents) *(~10 minutes)*

1. Who were the key influential people that needed to **approve** of doing SAIA at your facility? By this, I mean starting the SAIA process and also testing changes along the way.
   - What were their opinions about the SAIA intervention and the changes your team wanted to test? What made you feel that they were supportive or not supportive of the intervention?
   - How did their time availability and leave schedule influence your ability to do the SAIA intervention? Did this change over time? Why?
2. Who else was a leader or champion for SAIA at your facility? Outside of your facility?
   - What was their role specifically and how did they get this role? (e.g. appointed, volunteered)
   - What did they do that made you feel like they were a champion of SAIA?
   - How did participation in SAIA related to their regular job?

Finally, I want to ask you about **continuing** to do SAIA at your facility now that the study is finished. To clarify, this means using the SAIA tools as part of your day to day operations, as part of your program work, not as a research study. *(~10 minutes)*

1. What do you think about continuing SAIA at your facility? What would make it easy or difficult to do this? (CFIR domain and construct: inner setting: implementation climate: compatibility)
   - What structures, tools, or meetings did the study provide that were not part of your regular work? How do you think your work practices would need to change to still use these structures, tools, or meetings? (CFIR domain and construct: inner setting: implementation climate: compatibility)
   - What activities did the SAIA study team (Nancy, Geoffrey, Maina, and Dr. Gaitho) do and who would take on these duties now? (CFIR domain and construct: inner setting: implementation climate: compatibility)
   - After your experience with SAIA in a study setting, how would you change it to make it better for your facility specifically? (CFIR domain and construct: innovation characteristics: adaptability)
