# Supplementary material for: Providing “a beam of light to see the gaps”: determinants of implementation of the Systems Analysis and Improvement Approach applied to the pediatric and adolescent HIV cascade in Kenya
Source: Implement Sci Commun. 2022 Jul 16;3:73. doi: 10.1186/s43058-022-00304-3 (PMC9287987; doi:10.1186/s43058-022-00304-3)
Supplement: Supplementary file 2 — Additional file 2. SAIA-PEDS In-Depth Interview Question Guide. [file 43058_2022_304_MOESM2_ESM.docx]

**SAIA-PEDS In-Depth Interview Question Guide**

**Instructions:**

The following is a guide. Try to ask all the questions below in the order given, but it is more important to maintain the flow of discussion. Suggested probes have been included. Start with the following introductory script:

***************************************************************************************************************************************

*Hi, my name is ____________. Thank you for agreeing to participate in an interview today. I am interested in understanding your thoughts, experiences and opinions about doing the SAIA intervention in your facility. I will ask you questions that you are free to answer in any way you wish. Feel free to elaborate on any of your points. If a question is unclear to you, please feel free to ask me to explain it.*

*I would like to record the discussion so I don’t miss anything that you say. I will not include your names on any documents or in the recording. Your answers will be kept confidential, which means we will keep what you say private from others. Is it okay if I record our discussion?* [Wait for the participant to give verbal consent to recording]

*Before we start I would like to remind you that there are no wrong answers in this discussion. We are interested in knowing what you think, so please feel free to be open and share your point of view. We hope you can help us understand what did and did not work in the SAIA PEDS intervention so that we can make changes in the future. Your comments about what did not work are just as helpful as your comments about what did work. It is very important that we hear your opinion. You do not have to answer all the questions. If you want to stop the discussion at any time, just let me know.*

*As we get started, I want to remind us of the names of some tools so that we are all together in understanding. “****SAIA****” is an intervention that your facility participated in to improve HIV testing, treatment, and virologic suppression for children and adolescents. SAIA had 3 tools that you used. The first tool was the “****PedCAT”*** *[show picture of PedCAT], which showed your facility’s monthly data for HIV testing, linkage to care, ART initiation, viral load testing, and viral load suppression. The second tool was making “****flow maps”*** *[show picture of a flow map] of how patients move through your facility for services. The last tool was the process of testing small changes in clinic operations, sometimes called workflow modifications; we called this continuous quality improvement, or “****CQI****”. Together, SAIA used the* ***PedCAT****,* ***flow mapping****, and* ***CQI****.*

*Today, I will ask your opinions and thoughts about how your facility operates and a few questions about SAIA.*

***************************************************************************************************************************************

I would like to hear about the ways that **communication** happens at your facility, not just during the SAIA PEDS study.

1. Tell me about a time when you needed to work with others to solve a problem at your facility. (CFIR domain and construct: inner setting: networks & communication)
   - What was the problem and how did you choose who to talk to solve it?
   - What kinds of challenges did you face as a team and how did you communicate to resolve them?
2. Tell me about a time when you needed to work with others to solve a problem during SAIA PEDS. (CFIR domain and construct: inner setting: networks & communication)
   - What was the problem and how did you choose who to talk to solve it?
   - What kinds of challenges did you face as a team and how did you communicate to resolve them?
3. Can you describe your working relationship with leaders, like in-charges and supervisors, at your facility generally? How was your working relationship with them during the SAIA PEDS study? (CFIR domain and construct: inner setting: networks & communication)
4. Tell me about meetings at your facility. When do they happen? What is the typical agenda? Who usually attends? In your opinion, what about these meetings was helpful? What was not helpful? (CFIR domain and construct: inner setting: networks & communication)
5. Tell me about meetings at your facility related to SAIA PEDS. When did they happen? What was the typical agenda? Who usually attended? In your opinion, what about these meetings was helpful? What was not helpful? (CFIR domain and construct: inner setting: networks & communication)

I would like to hear about the ways that **changes** happened at your facility during the SAIA PEDS study.

1. Can you share an example of a new idea or change tried during SAIA PEDS? How was this, and other, new ideas and changes during SAIA PEDS received at your facility? How were the new ideas and changes used to make improvements in your facility? (CFIR domain and construct: inner setting: culture)
2. Do you feel like you can try new things to improve your work processes generally in your facility? Why or why not? (CFIR domain and construct: inner setting: implementation climate: learning climate)
   - Do you feel like you have the time and energy to think about ways to improve things generally in your facility?
   - How was this similar during SAIA PEDS? How was it different during SAIA PEDS?
   - Did you feel appreciated by your supervisor for the role you played in SAIA PEDS? How and why?
3. Do you think your supervisor will consider your role in the SAIA project in their evaluation of you? (CFIR domain and construct: inner setting: implementation climate: organizational incentives & rewards)

Now let’s talk about **leaders and champions** at your facility. We are interested in all kinds of leaders, the formally appointed leaders of the facility, the informal leaders in your facility, champions for specific patient populations or activities, leaders in your local area that are not part of your facility but have influence here, or even just the informal leaders that someone turns to at your facility when they have an important decision to make. (CFIR domain and construct: process: engaging opinion leaders, formally appointed implementation leaders, champions, external change agents) *(~5 minutes)*

1. Who were the key influential people that needed to **approve** of doing SAIA at your facility? By this, I mean starting the SAIA process and also testing changes along the way.
   - What were their opinions about the SAIA intervention and the changes your team wanted to test? What made you feel that they were supportive or not supportive of the intervention?
   - How did their time availability and leave schedule influence your ability to do the SAIA intervention? Did this change over time? Why?
2. Who else was a leader or champion for SAIA at your facility? Outside of your facility? (NOTE: Any cadres can fit this role)
   - What was their role specifically and how did they get this role? (e.g. appointed, volunteered)
   - What did they do that made you feel like they were a champion of SAIA?
   - How did participation in SAIA related to their regular job?

Now let’s talk about **goals and feedback** in your facility

1. How did your facility or clinic set goals for SAIA PEDS? (CFIR domain and construct: inner setting: implementation climate: goals & feedback)
   - How were these goals communicated in this facility? To whom were they communicated? Can you give an example?
2. Tell me about how feedback was given at your facility to individuals about their performance, either during routine supervision or during performance appraisal. (CFIR domain and construct: inner setting: implementation climate: goals & feedback)
   - How do you get feedback? Who gives it? What is the format? How often do you get feedback?
   - How helpful is this feedback and how could it be improved?
   - How was this process similar or different for SAIA PEDS?

Finally, let’s talk about **using the SAIA intervention** in your facility

1. Generally, how do you feel about using the SAIA intervention at your facility in the future? How confident do you and your colleagues feel about using the SAIA intervention going forward? What makes you feel this way? What would you need to feel more confident? (CFIR domain and construct: characteristics of individuals: self-efficacy)
